# Two-component systems as potential therapeutic targets in *Neisseria* pathogenesis

**DOI:** 10.1080/07853890.2025.2599592

**Published:** 2025-12-09

**Authors:** Tiyasa Haldar, Sunil D. Saroj

**Affiliations:** Symbiosis School of Biological Sciences, Symbiosis International (Deemed University), Pune, Maharashtra, India

**Keywords:** Two-component systems, *Neisseria meningitidis*, *Neisseria gonorrhoeae*, antimicrobial resistance, virulence, therapeutic targets

## Abstract

**Introduction:**

*Neisseria meningitidis* and *Neisseria gonorrhoeae* are obligate human pathogens that cause meningitis and gonorrhea, contributing substantially to global morbidity. Although vaccines are available for select meningococcal serogroups, no licensed vaccine exists for gonorrhea, and rising antimicrobial resistance has further complicated treatment. These challenges highlight the need for alternative therapeutic strategies. Two-component systems (TCSs) are major regulatory mechanisms in bacteria, enabling environmental sensing and adaptive responses essential for survival and pathogenesis.

**Discussion:**

Despite possessing relatively few TCSs, *Neisseria* relies heavily on these systems to coordinate virulence-associated functions. The MisS/MisR system regulates capsule synthesis, lipooligosaccharide modification, and resistance to antimicrobial peptides. NtrX/NtrY and NarQ/NarP modulate respiratory adaptation under oxygen-limited conditions, while pilus-associated systems govern adhesion, motility, and tissue colonization. TCSs also influence antimicrobial resistance by controlling efflux pumps, envelope homeostasis, and stress responses, thereby supporting persistence and multidrug resistance. Their dual involvement in pathogenesis and drug resistance underscores their therapeutic relevance. Several TCS-regulated proteins, such as AniA and type-IV pilus subunits, have shown promise as vaccine antigens. Advances in histidine kinase inhibitors and structure-based screening further expand opportunities to target TCSs as antimicrobial interventions.

**Conclusions:**

TCSs serve as essential regulators of virulence and antimicrobial resistance in *Neisseria* spp. Their conserved and limited repertoire makes them appealing targets for drug and vaccine development. TCS-directed strategies offer a promising route to complement existing therapies and address the growing challenge of meningococcal and gonococcal infections.

## KEY MESSAGES

1. TCSs regulate capsule, pili, and respiration in *Neisseria*, enabling adaptation and survival within humans. Their limited number makes them essential for virulence and disease.
2. TCSs promote antimicrobial resistance by controlling efflux pumps, envelope integrity, and responses to host peptides, driving persistence and multidrug resistance in pathogenic *Neisseria*.
3. Targeting TCSs offers innovative therapeutic strategies. With limited vaccines and rising resistance, TCS inhibitors may provide effective alternatives for treating meningococcal and gonococcal infections.

## 1. Background

*Neisseria meningitidis* and *Neisseria gonorrhoeae* are gram-negative diplococcal commensals that colonize the human body. While most *Neisseria* species are non-pathogenic commensals, *N. meningitidis* and *N. gonorrhoeae* are obligate human pathogens that cause meningococcal meningitis and gonococcal sexually transmitted infections, respectively, with significant morbidity and mortality rates in both developed and developing nations. Humans are the sole hosts of both the species. Thus, bacteria have developed unique adaptive mechanisms to survive in human host defense mechanisms, acquire nutrients from the host environment, and compete with the microbiome [1]. Gonococcal infection imposes a significant increase in the global burden of STI, with 87 million cases annually. In the United States, an estimated 111% increase in the total number of cases was observed between 2009 and 2020, while in Europe and Australia, 218% and 127% increases in total cases were reported from to 2009–2018 and to 2012–2019 respectively [2,3]. In addition, in high-income nations, the incidence is high in transgender persons, sex workers, racial/ethnic minorities, and indigenous peoples. Furthermore, a rapid increase in antimicrobial resistance in *Neisseria* has been reported worldwide [4]. Therefore, there is an urgent need to develop effective treatments that target the physiological mechanisms of the bacteria [5]. The first successful vaccine against meningococci was composed of pure polysaccharides from four major serogroups [6]. However, the polysaccharide composition used for vaccines mimics human N-acetyl neuraminic acid, which can develop autoantibodies. Also, BEXSERO and TRUMENBA were recently approved against the group B meningococci. However, still there is a need to find therapeutic target against *Neisseria* infection. In addition, there is currently no licensed vaccine for gonorrhea.

*N. meningitidis* and *N. gonorrhoea* asymptomatically colonize the mucosal surface of the upper respiratory tract or the genital tract of humans, but occasionally invade the epithelial barrier and reach the bloodstream, causing severe inflammation. However, the specific mechanism and factors involved in the transition from the asymptomatic to the symptomatic state of *Neisseria* sp. to cause invasive disease remain unclear. Several studies have suggested that host immune responses, genetic factors, and environmental factors may play a role in this transition, but the precise interactions and contributions of these factors remain elusive [7]. However, previous studies have demonstrated that different factors related to both the host and pathogen can contribute to alterations in bacterial virulence. For example, different host environmental stimuli, such as the availability of nutrients and pH, can influence bacteria to show different phenotypic changes that can lead to the development of symptomatic infections [8]. To sense these signals, pathogenic bacteria have an important machinery known as a two-component system that helps them to identify external signals and regulate different virulence properties to successfully colonize [9]. More than 4000 two-component systems (TCSs) have been identified across 145 sequenced bacterial genomes, indicating the critical role of TCSs in the environmental adaptation of bacteria. Two-component systems (TCSs) are ubiquitous signal transduction pathways in prokaryotes that allow organisms to recognize signalling molecules and regulate physiological responses as an adaptation to environmental stimuli [10]. Bacterial TCSs are a specific type of regulator that perceives a wide range of signals that regulate the transcription of a large number of genes. TCSs are made up of two multi-domain proteins, a response regulator (RR) protein and a sensor histidine kinase (HK) domain that help to recognize and react to particular environmental cues such as temperature, pH, availability of nutrients, quorum signals, and antibiotics etc [11]. After recognizing the signal, the sensor kinase protein phosphorylates the response regulator, which modifies gene expression or other physiological functions and enables bacteria to respond to stress conditions. The host microenvironment plays an intricate role in the expression of TCSs by providing a unique set of challenges and opportunities for pathogenic bacteria [12]. Moreover, a significant positive correlation between the number of TCSs and genome size was established, which suggests that bacteria with larger genomes have a greater number of TCSs. Unlike other pathogens, *Neisseria* sp. has a smaller genome and fewer two-component systems, with only six projected pairs owing to its constrained habitat. These functional two-component regulatory mechanisms have been found to play crucial roles in physiology, suggesting that they are significantly involved in controlling the virulence and antimicrobial resistance of *Neisseria* sp.

## 2. Two-component systems present in pathogenic *Neisseria* sp

*Neisseria meningitidis* and *Neisseria gonorrhoeae* possess a relatively small number of TCSs compared to other pathogens; each system plays a highly specialized and non-redundant role in regulating virulence, metabolism, and antimicrobial resistance. These systems coordinate gene expression in response to environmental cues such as envelope stress, oxygen limitation, and nitrosative stress, thereby enhancing bacterial survival during colonization and infection (Table 1).

**Table 1.** Factors governing the expression of two component systems in *Neisseria* sp. and their role in stress response.

TCSs	Signals	Functions	Stress response and virulence	References
MisS/MisR	Alteration in the membrane	Capsule synthesis, LPS modification	Response in envelop stress	[13]
NtrX/NtrY	Redox potential	Anaerobic respiration	Survival in oxidative stress	[14]
NarQ/NarP	Presence of nitrite	Nitric oxide denitrification	Survival in oxidative stress	[15]
Nps/Npa	High pilin signal	Transcription of pilin subunit	Attachment to host cells	[16]
PilA/PilB	Environmental stimuli	Motility, host cell attachment	Attachment to host cells	[17]

Understanding the specific TCSs encoded in *Neisseria* is crucial to deciphering their contribution to host-pathogen interactions and to identify potential therapeutic targets. The following sections discuss the major characterized TCSs in *Neisseria*, highlighting their molecular mechanisms, physiological roles, and links to virulence regulation.

### 2.1. MisS/MisR

In *Neisseria* the presence of a limited number of TCSs suggests that TCSs are involved in maintaining bacterial physiology and virulence. MisS/R is a two-component regulatory system found in both gonococcus and meningococcus [18,19]. In *N. gonorrhoeae* MisS/R was found to be a homolog of the CpxRA system in *Escherichia coli*. This system senses envelope stress and regulates the expression of genes encoding envelope-localized proteins [20]. Kandler et al. reported that the MisR protein plays a necessary role in the *in vivo* survival of gonococci by providing membrane stability. MisR in gonococci has also been found to impart antimicrobial resistance in *N. gonorrhoeae* [21]. Additionally, gonococcal MisR and MisS proteins were found to be required for survival during heat shock stress and cervicovaginal colonization in mice [18]. The MisS/R system of *N. meningitidis* was previously characterized and named based on its role in regulating the meningococcal inner core structure of lipopolysaccharides [13]. The MisS/R two-component system is autoregulatory. Upon sensing environmental signals, MisS gets phosphorylated in the presence of ATP and shows phosphotransferase activity to pass the signals to MisR. MisR then directly binds to specific motifs present in the upstream region of *misRS* promoter, which activates transcription [22]. Lipopolysaccharide (LPS) plays a major role in meningococcal pathogenesis and as an inflammatory mediator. In addition, LPS is involved in the colonization, serogroup determination, and antimicrobial susceptibility of bacteria. In addition, LPS is considered a potential vaccine candidate as a host immune target [23–25]. Therefore, it will be interesting to develop an attenuated vaccine targeting the meningococcal inner core protein MisS/R system, which might have higher efficacy than the conventional one [26].

**Figure 1.**
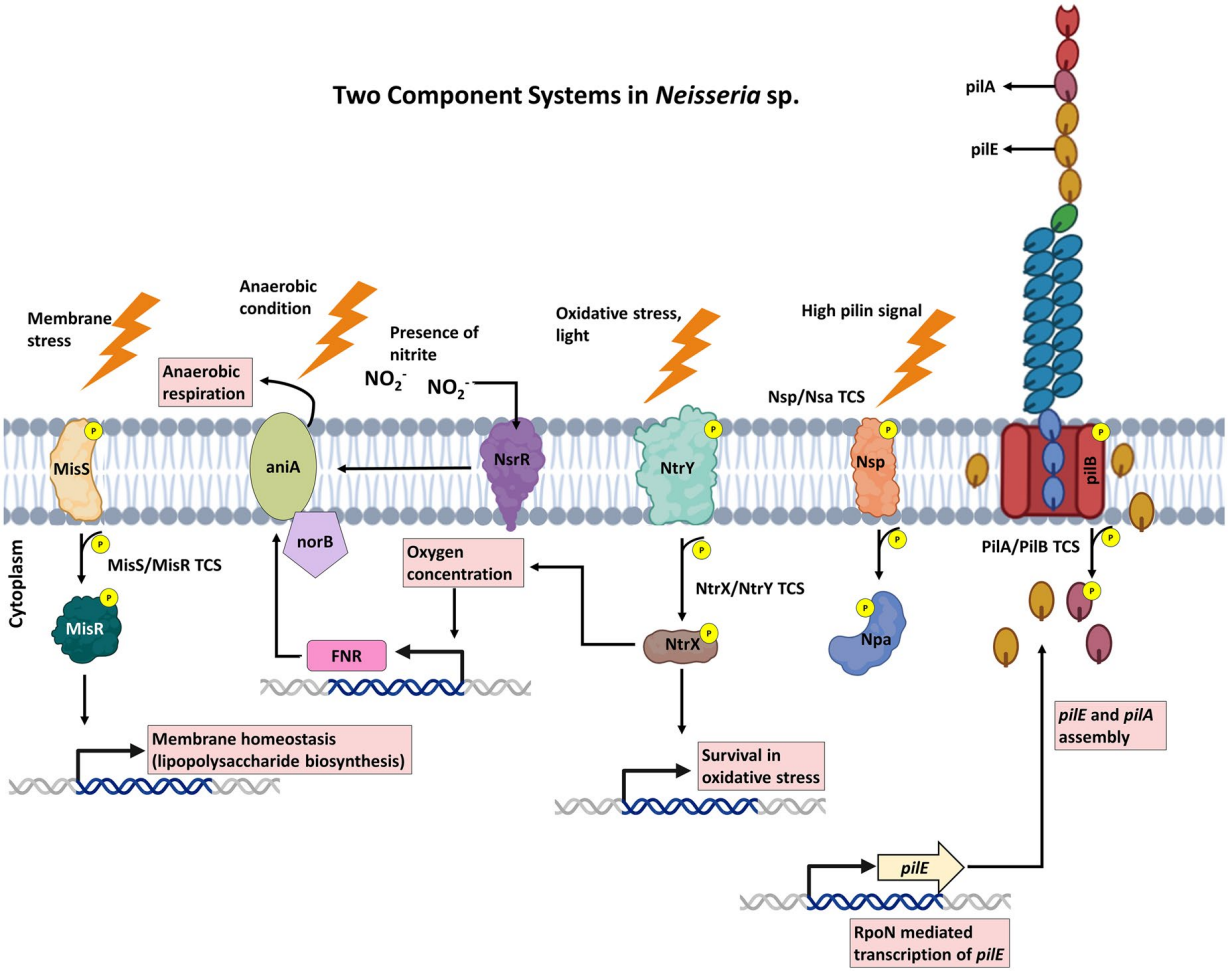
Two-component systems regulating stress responses in *Neisseria meningitidis* and *N. gonorrhoeae.*

The MisS/R system is homologous to the CpxR/CpxA system in different gram-negative bacteria. CpxR and CpxA system regulates the bacterial response to envelop stress where CpxA gets autophosphorylated and transfer the phosphate group to CpxR. CpxR regulated the expression of envelop protein folding. Moreover CpxR decreases the expression of genes involved in envelop localized proteins to relax the production of nonessential proteins during stress condition [18,20]. In addition, the MisS/R was found share functional similarity to the PhoP/Q system in *Salmonella* [27]. In *Salmonella*, the PhoP/Q system has been well studied and found to regulate multiple virulence genes in response to different environmental changes. Johnson et al. showed that the meningococcal MisS/R knockout mutant exhibited many similarities with the *Salmonella* PhoP mutant. Some of the traits were that the mutant was unable to grow in a magnesium-limiting condition, as observed in *Salmonella*, was highly sensitive to human neutrophil-derived defensins, and lost magnesium-mediated gene regulation. However, in meningococci mutants, there were different responses to acidic pH compared to *Salmonella* mutants [28]. MisS/R system is found to be required for the virulence of *N. meningitidis* as it was observed that, in meningococci 78 genes responsible for protein folding, iron acquisition, and other virulence genes were regulated by MisR/S in murine infection model. MisS/R was shown to regulate different genes either by directly binding to the promoter region of genes or by indirectly modulating protein folding pathways and other cellular mechanisms (Figure 1). In addition, the system may be involved in regulating the expression of *lptG* gene, which encodes o-3 linked glucosyltransferase. Decreased expression of o-3 linked glucosyltransferase results in increased level of o-3 linked glucose moiety on lipooligosaccharide (LOS). Consequently, modification of LOS influences the bacteria to become resistant towards complement-mediated killing. Moreover, this system negatively regulates different chaperone genes, *dnaJ*, *clpB*, and *fkpA*, which are involved in protein folding [22,29].

Capsular polysaccharides and outer membrane proteins are major virulence determinants in both *N. meningitidis* and *N. gonorrhoeae.* Meningococcal capsule biosynthesis occurs under the regulation of *cps* locus, which includes a promoter region located between capsule biosynthesis and the capsule transport operon [30]. The intergenic region of the *cps* locus is controlled by the MisR/S system. MisR negatively regulates capsule formation by directly binding to the divergent promoter region of the capsular gene [31]. On the other hand, in gonococci OmpA like proteins are diverse groups of outer membrane protein plays important role in gonococcal adhesion, invasion and survival in lower genital tract [32]. Previously, it was demonstrated that the transcription of *ompA* is regulated by MisR, as OpmA was found to be a part of the MisS/R regulon. The MisR response regulator protein binds to a specific DNA sequence on the upstream promoter region of ompA to initiate transcription [33]. Consequently, the increased outer membrane protein and capsular polysaccharide promote the survival of gonococci and meningococci in the host. However, a particular environmental signal influencing TCS and its regulatory sequence is yet to be studied [34].

### 2.2. NtrX/NtrY

Cellular respiration is essential for survival and energy production in all organisms. In the host environment, bacteria encounter varying concentrations of oxygen and electron acceptors. Therefore, in bacteria, several enzymes have evolved to survive under such diverse conditions. In an oxygen-limiting environment, the NtrX/Y TCS plays an important role in the survival of *N. meningitidis* by participating in nitrogen metabolism. NtrY acts as a sensory kinase consisting of membrane-bound proteins with three domains: the cytoplasmic HAMP protein, PAS, and histidine kinase domains. The PAS domain is a well-known regulatory protein found in bacteria that recognizes oxygen levels, redox potentials, and light [35]. In contrast, NtrX is a response regulator consisting of four different classes of NtrX regulator proteins [29], and *Neisseria* cytochrome cbb3 oxidase, a crucial respiratory enzyme, was found to participate in bacterial virulence and survival under oxidative stress [36]. This enzyme catalyses electron transfer from cytochrome c to oxygen molecules. Under oxygen-limiting conditions, enzyme activity is reported to be the highest [37]. In *Rhodobacter capsulatus*, cytochrome c oxidase activity is regulated by a RegBA TCS [38]. Although *Neisseria* sp. there is no TCS resembled RegBA in *Neisseria* sp., one of the TCS present in this bacterium was found to be the ortholog of the NtrX/Y TCS, a P_II_ type signal transduction protein that is involved in nitrogen fixation and other metabolic activities [39].

NtrX has been observed to regulate the function of different respiratory enzymes and the adaptive ability of bacteria. In contrast, in oxidase-negative bacteria such as *Enterobacteriaceae* ArcAB TCS modulates survival in a redox environment. In addition, in a mouse model of bacteria, the ArcAB TCS was observed to be required for the survival of *Hemophilus Influenzae*. In the NtrX mutant *of N. gonorrhoeae* similar response was observed with altered oxygen concentration. Moreover, *norB* and *aniA* encode two crucial respiratory complexes involved in denitrification, are dependent on NtrX.

### 2.3. NarQ/NarP

The NarQ/P TCS was found solely in β- and γ-proteobacteria. To date, three other NarQ family proteins have been identified in *E. coli* and *Pseudomonas stutzeri* [40]. In *N. meningitidis* NarQ can sense the presence of nitrite as an environmental signal, and NarP acts as a response regulator that regulates the expression of genes involved in the reduction of nitrite to nitrous oxide. *N. meningitidis* can survive oxygen starvation by reducing nitrite to nitrous oxide. This process is catalyzed by nitrite and nitric oxide reductase enzymes encoded by AniA, an outer membrane protein, and NorB. Fumarate and nitrate reductase regulator protein (FNR) is an oxygen-sensing transcription factor that is essential for the activation of the AniA promoter region [41,42]. Moreover, in the presence of nitrite, the complete activation of the entire operon is regulated by the two-component NarQ/P system. From microarray studies, five genes were identified at the *narP* binding site. Among these three genes, *aniA*, *norB* and *narQ* were linked to the truncated denitrification pathway in *N. gonorrhoeae*. In addition, gonococcal NarP shares sequence similarity with the *E. coli* NarP-binding site. However, the gonococcal NarQ/P TCS was insensitive to nitrate/nitrite. Furthermore, instead of NarQ-NarP, the NsrR protein, an IscR transcription factor, was observed to be essential for nitrite-induced *aniA* expression [15,43]. Other studies have reported that in gonococci, the expression of *aniA* is induced by nitrite but not by nitrate. The difference is because there is considerable sequence variation between the *Neisseria* protein and other enterobacterial proteins, as only 10 out of 18 residues were conserved in the gonococci homologue protein. Because of these significant sequence dissimilarities with their *E. coli* counterparts, they cannot fully substitute for each other, suggesting differences in RNA polymerase interactions [44]. Thus, the presence of alternative mechanisms to adapt to different environments suggests that transcriptional activation to tightly regulate gene expression is important in *N. meningitidis*. The ability to quickly adapt to an oxygen-deficient environment is crucial for pathogenic *Neisseria* sp. to infect the mucosal surface and proliferate in inflammatory exudates [45].

### 2.4. PilA/PilB

Pili are one of the most important components of the bacterial structure and play an important role in motility, cell signalling, host cell attachment, biofilm formation, exchange of genetic material *via* conjugation, and other activities. It is also one of the major virulence factors for host-pathogen interaction since initial adherence of bacteria to the host cell is required to cause the spread of infection. Type IV pili (Tfp) are widely present in gram-negative bacteria. Tfp is involved in motility and adhesion, but it can also aid bacteria in extracellular DNA absorption, allowing them to acquire numerous virulence features such as resistance to antibiotics [46]. Pilin is the major subunit of the pilus and the chromosomal locus PilE controls the transcription of pilin units. In gonococci, two closely related genes, *pilA* and *pilB*, regulate the transcription of pilin units. PilB acts as a cytoplasmic histidine kinase sensor protein, and PilA is a response regulator in the M-terminal region. Furthermore, PilA and PilB share amino acid sequence homology with two-component system proteins. PilA is also associated with the eukaryotic secretory protein domain, known as the SRP receptor and SRP 54, and the C-terminal putative GTP-binding site. Mini-transposon insertion and other phenotypic and genotypic studies have revealed that PilA is a DNA binding response regulator involved in different physiological functions in bacteria [17].

### 2.5. Nps/Npa

As mentioned earlier, in *Neisseria* the *pilE* is a structural subunit anchored to the cell membrane by complex components in Neisseria. *pilE* transcription is regulated by a two-component system named Nps/a. Npa is an activator found to be required for RpoN mediated transcription of pilE [47]. NPA acts as a positive regulator of pilE transcription. Furthermore, the Npa protein has sequence homology with the response regulator of a TCS with a N N-terminal receiver domain and C-terminal output domain. Additionally, the upstream of Npa was found to be an open reading frame, named as *Neisseria* pilus sensor (Nps), with amino acid sequence similarity found in typical histidine sensor kinase proteins [16]. Previously, it was reported that the transcriptional regulation of *pilE* is different in pathogenic *Neisseria* and commensal *Neisseria*. In pathogenic *Neisseria* the transcription of the pilE subunit is regulated by the integration of the host sigma factor RpoD, also known as σ^70^, and repressor proteins RegF and CrgA. In commensal *Neisseria* the transcription is regulated by sigma factor RpoN (σ^54^). Hence, this regulatory difference indicates that switching from an RpoN- to an RpoD-dependent mechanism of *pilE* transcription in pathogenic *Neisseria* is a result of evolution as pathogenic *Neisseria* diverged from commensal *Neisseria*. In experimental studies, it was observed that Nps is required for Npa function, which in turn regulates pilE transcription of *pilE* [16].

## 3. Two component system and their role in antimicrobial resistance (AMR)

Different environmental changes, such as the presence of antimicrobials, can influence the expression of TCSs, which may develop AMR. In addition, TCS-mediated alterations in bacterial physiology can contribute to AMR in response to other environmental stimuli. Human cationic antimicrobial peptides (CAMPs) are produced as part of an innate immune response during different microbial infections. Therefore, host bacteria regularly encounter different cationic antimicrobial peptides, which can lead to the development of intrinsic and inducible resistance mechanisms in bacteria. These resistance mechanisms include modification of outer membrane permeability [48], increased expression of drug efflux pumps, production of membrane-bound proteases that degrade CAMPs [44], and alteration of cell surface proteins, which can change the interaction of CAMPs with lipopolysaccharides and lipooligosaccharides [49]. In *P. aeruginosa* the two-component systems PhoP/Q and PmrA/B control lipid A modification, which confers resistance towards CAMPs and polymyxin B [50]. In *N. meningitidis* two mechanisms play a role in the development of resistance to antimicrobial peptides: modification of lipid A by substitution of phosphoethanolamine at the head group and expression of efflux pumps to elute CAMPs [51]. Tzeng et al. showed that the mutation in the MisS/R two-component system in *N. meningitidis* resulted in the loss of phosphoethanolamine from the inner core HepII residue of lipopolysaccharide, which leads to the increased sensitivity of the bacteria toward CAMPs and polymyxin B [13]. In gonococci, MisR is required for constitutive and inducible levels of gonococcal resistance to CAMPs. Moreover, MisS/R regulates the redox potential and integrity of the bacterial cell envelope. Deletion of this TCS leads to misfolding of proteins in the cell envelope, which makes the cell membrane more permeable to antibiotics [21].

Efflux pumps are active transporter proteins that maintain bacterial homeostasis by the expulsion of toxic molecules, such as antibiotics. The increased expression of efflux pumps is one of the major reasons for the development of multiple drug resistance (MDR) in bacteria. Efflux pumps are differentiated based on their energy utilization sources. The ATP-binding cassette (ABC) family of efflux pumps functions by utilizing the energy from hydrolyzed ATP, whereas other superfamilies, such as resistance nodulation and cell division (RND), and multidrug and toxic compound extrusion (MATE), depend on the proton motive force provided by the H^+^ and Na^+^ electrochemical gradient. In *N. gonorrhoeae* NorM, a MATE efflux pump was reported to protect bacteria by exporting cationic antimicrobials and shielding them from reactive oxygen species. NorM interacts with the substrate depending on ionic strength and hydrogen bonding. Certain conserved amino acid sequences have also been found to participate in these interactions. Therefore, the conformational site in the NorM efflux pump plays an important role in binding efficiency to the antibiotic-binding pocket. In *Acinetobacter baumannii* AdeRS two-component system regulates the function of the RND-type efflux pump adeABC. *adeS* senses saline stress as well as the presence of pentamidine, and regulates the expression of efflux pumps. Therefore, there might have a significant influence of TCSs behind the regulation of efflux pump function in *Neisseria* sp.

## 4. Two component system as a potential target for antimicrobial therapy

**Figure 2.**
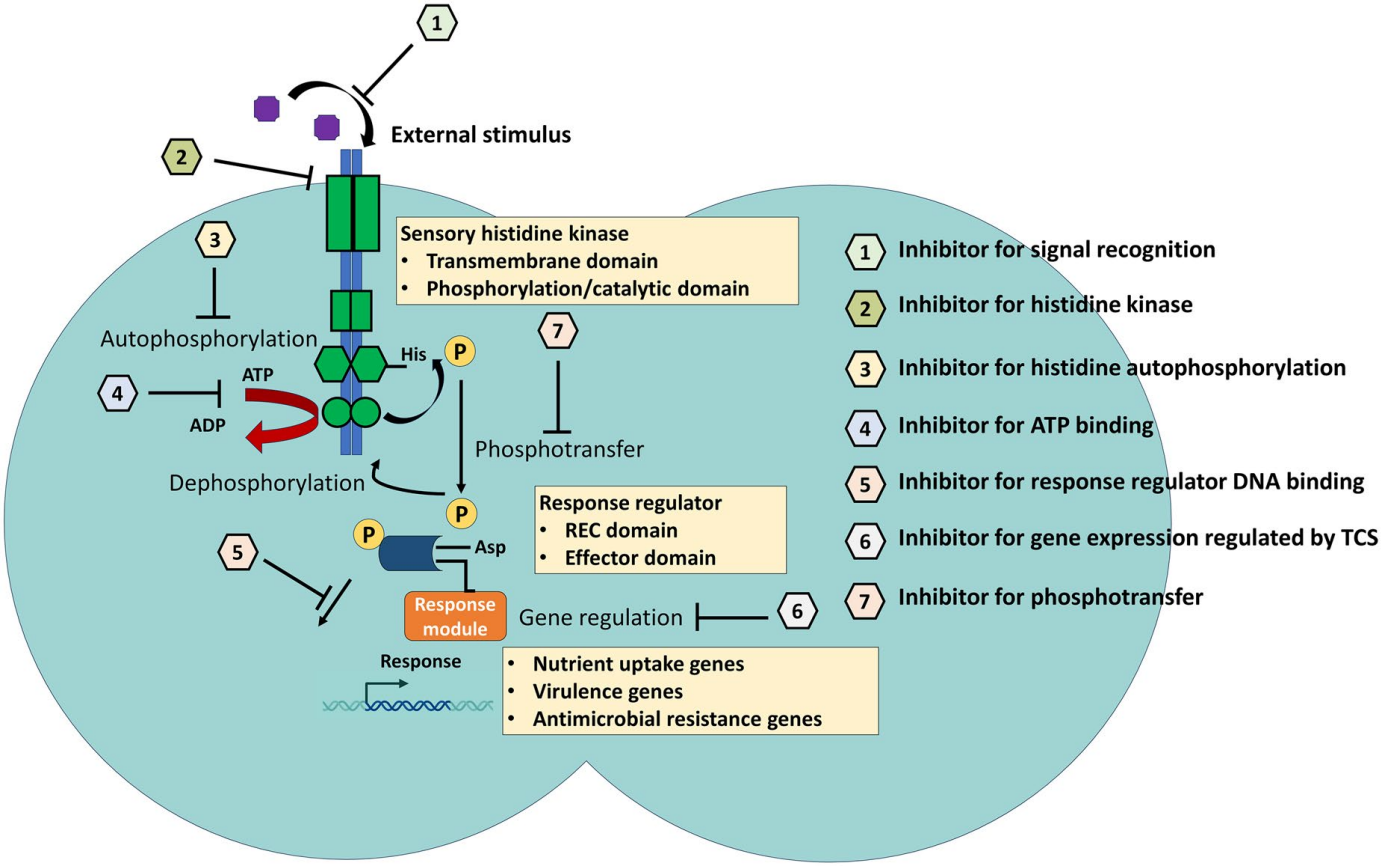
Potential therapeutic strategies to inhibit two-component systems in pathogenic *Neisseria*. Different inhibitors can potentially disrupt the two-component system function which further attenuate the virulence.

In the present scenario, considering different factors such as the global rise in AMR due to rapid evolution in bacteria, co-morbidity with autoimmune diseases such as HIV, and bacteria forming persister cells that remain in the dormant stage for a prolonged time, there is an urgent need for novel therapeutics to cure bacterial infections. Consequently, fundamental knowledge of microbial pathogenesis and cell signalling pathways, in combination with modern drug discovery, can be a new approach to develop new therapeutics. The virulence factors and regulatory systems responsible for modulating their expression are considered the major therapeutic targets for different pathogenic bacteria. As previously mentioned, TCSs play a pivotal role in regulating virulence factors in bacteria. Therefore, TCS may be a potential target for antimicrobial drugs. Conventional antimicrobial drugs often target specific bacterial proteins that are involved in essential cellular processes. However, this approach can lead to the development of antimicrobial resistance in the bacteria. A drug that targets the TCS of bacteria could be highly efficient because these drugs can directly disrupt the upstream regulatory mechanisms that are involved in the physiological process of the organism. Considering the potential involvement of TCSs in bacterial pathogenesis, the pharmaceutical industry is currently focusing on the development of suitable inhibitors of the signal transduction process. The molecular mechanism of signal transduction *via* a two-component system in bacteria has been well-studied. Targeting histidine kinase sensor proteins has been reported to be an effective strategy (Figure 2). However, one potential limitation of targeting the histidine kinase is that in eukaryotes and bacteria, the kinase protein shows a high degree of sequence similarity. Moreover, the ATP binding site, including the chaperone Hsp90, is a crucial protein found in multiple organisms that plays crucial physiological roles. Previously, it was found that the catalytic domain and receiver protein of histidine kinase and the response regulator share significant sequence homology [52]. Thus, designing a single drug targeting any of these conserved sequences present in both the kinase and response regulator domains can block multiple TCSs simultaneously. This might increase the chances of developing defense against any kind of mutation in the molecule that affects drug affinity to the target site. However, some TCSs are not essential for bacterial survival in laboratory condition, but they can improve the bacterial stress adaptation and helps in increasing antimicrobial resistance [53]. Therefore, targeting essential and non-essential TCSs is a critical consideration in developing antimicrobial therapeutics. While inactivation of essential TCSs involved in bacterial viability drives rapid development of antimicrobial resistance due to its high selective pressure. On the other hand, targeting non-essential TCSs that mostly govern the virulence offers a slower acquisition of resistance [54]. For example, in *S. aureus* AgrACDB TCS is not essential for the bacterial survival but plays very important role in different virulence characteristics. Targeting Agr system can be more effective as a therapeutic target [55].

As noted above, *Neisseria* can grow anaerobically *via* a two-step denitrification process, under the regulation of the NarP/Q system. *aniA* is a crucial gene present in the NarP-binding site. *aniA*, which encodes a copper-containing enzyme that converts nitrite to nitric oxide. Furthermore, nitic oxide was reduced by *NorB*. In contrast, nitrate reductase and cytochrome c oxidase promote the survival of *Neisseria meningitidis* from reactive oxygen and nitrogen species. Shewell et al. demonstrated that immunization with different glycoforms of the AniA protein resulted in a strong humoral immune response towards basal monosaccharides in a rabbit model. Furthermore, immunization with truncated *aniA*, lacking the complete glycosylated C-terminal region, showed that the development of antibodies against AniA is independent of glycosylation. As *Neisseria* expresses AniA on the cell surface, in a whole-cell experiment, antiserum against AniA completely inhibited the function of nitrite reductase. Therefore, recombinant aniA is a potential vaccine antigen against pathogenic *Neisseria* [56]. NtrX system in *N. gonorrhoeae* controls the expression of *aniA*, *norB* and cytochrome peroxidase during biofilm formation [57]. In other bacteria with NtrX/Y, inhibition of the histidine kinase unit by closantel attenuates bacterial evasion from immune clearance by lysosomes [58].

During *Neisseria* infection, type IV pili mediate endothelial attachment, triggering vascular damage. Thus, compounds that inhibit the adhesion of bacteria to host cells are a good choice for drugs. Neisseria pili of *Neisseria* is a major virulence factor. TCS PilA/B regulates major pilin units in bacteria. The pilin protein is composed of a complex protein machinery consisting of PilD, PilE, PilF, PilM, PilN, PilO, PilT, and PilQ subunits. PilE is the major pilin subunit, and PilF is involved in pili elongation. The PilT is involved in retraction. Aubey et al. identified different signalling pathway activators and inhibitors that inhibit Tfp assembly, which in turn prevented microcolony formation of *N. meningitidis* on the cellular surface. These compounds inhibited PilF ATPase activity to stop the pili extension process, but they were unable to block pilus retraction. This causes rapid loss of pili from the bacterial cell surface, leading to the inhibition of pilus-mediated attachment to the cell surface. Moreover, these molecules are highly selective to PilF, as they do not interfere with the structural similarity with PilT. In addition, *N. gonorrhoeae* showed similar sensitivity to these compounds. Therefore, these small molecules can be applied to a broad range of pathogenic *Neisseria* sp [59]. Thioridazine and trifluoperazine could inhibit the Type IV pili function including twitching motility, adhesion capability and aggregate formation in *N. meningitidis* [59]. Trifluoperazine targets inhibit the activity of Na^+^ pump of NADH-ubiquinone oxidoreductase complex which plays important role in energy production. Therefore, perturbation of energy production leads to the reduction in piliation on the bacterial cell surface [60]. Also, in Group A Streptococcus (GAS) pilus is one of the most important factors governing the virulence. Also, gas pili major component is a key antigen that can be considered as a vaccine candidate as the pilus structure act as highly effective antigen delivery platform by enhancing the B cell accessibility and better antigen presentation [61]. Previous studies were focused on the utilization of pilus tips to successfully elucidate the mucosal and systemic antibody response by dendritic cell activation. However, limitation in structural stability and amplification of pilus tip enhanced the risk of proteolytic degradation of the antigen. As opposed, the alternative strategy to utilize the entire pilus backbone showed more stable backbone with strong covalent bond formation resulting multimerization and better amplification of antigen [62]. Yet, the lack of specificity can be a major drawback of the pili-based vaccine development. In case of *Neisseria gonorrhoeae,* it was reported that 100 to 112 mg of pili vaccine has significant response human volunteers with enhanced phagocytosis of the pathogen by human polymorphonuclear leukocytes [63]. Although, the vaccine was able to interfere with the colonization of gonococci to human cells, the main pilus subunit undergoes rapid antigenic variation [64]. Also, in other studies it was observed that a strain can evolve to present altered pilin expression and the immune sera against the antigen from a particular strain’s pilin was unable to hinder with the adhesion of another stains [65]. Therefore, pilin as a stand-alone vaccine component possess limitations as a potential therapeutic. However, further studies to identify the conserved pilin region can show some advances.

To develop new drugs targeting TCSs, different approaches can be considered. First, structure-based virtual screening (SBVS) should be performed using complex and large databases. This process enables the screening of potential inhibitors with structures that have been reported to have antimicrobial activity [66]. Subsequently, with the help of molecular docking analysis, the modelling refinement of the TCS protein structures can be studied. This may make it easier to identify the compounds using the SBVS technique. The structural data obtained from these proteins can also be utilized to pinpoint the chemical gaps and binding sites that permit interactions with potential chemicals, thereby improving the prediction of new inhibitors.

### 4.1. Targeting bacterial histidine kinase - potentials and challenges

Although most HK inhibitors have been studied in non-Neisserial pathogens, these findings provide a useful framework for evaluating the therapeutic potential of TCSs in *Neisseria*. HKs are highly conserved across Gram-negative bacteria, including *N. gonorrhoeae* and *N. meningitidis*, and regulate clinically important traits such as oxidative-stress adaptation, epithelial adherence, biofilm formation, and antimicrobial tolerance [67]. Given the rising prevalence of cephalosporin-resistant *N. gonorrhoeae*, targeting HK-mediated signaling offers an attractive anti-virulence or anti-resistance strategy.

Structurally, Neisserial HKs contain conserved ATP-binding (CA) and histidine phosphorylation (HisKA) domains similar to those in other pathogens, including the druggable Bergerat fold [68]. Early ATP-competitive inhibitors, such as diaryl-pyrazoles derived from Hsp90 scaffolds, demonstrated that these pockets can be targeted, although selectivity remained a challenge. More recent work has focused on inhibitors of the HisKA domain, which may offer greater specificity by engaging residues equivalent to the conserved His391 found in Neisserial systems such as MisR/MisS and NarQ/NarP [69].

In a landmark study, Wilke et al. screened over 53,000 compounds and identified molecules that competitively bind the ATP pocket of HK853 (*Thermotoga maritima*), VicK (*Streptococcus pneumoniae*), and CheA (*Escherichia coli*). Luteolin inhibited HK853 by occupying the ADP-binding pocket, but its broad activity against fatty acid biosynthesis and VEGF signaling limited its therapeutic utility [70]. Similarly, benzothiazole derivatives exhibited antibacterial activity but were cytotoxic at clinically relevant concentrations. Although candidates like luteolin lacked specificity, their binding profiles illustrate the feasibility of designing inhibitors that could be adapted for Neisserial HKs. Additional scaffolds, including benzothiazoles and thiazolidinones, are summarized in Table 2 and provide starting points for Neisseria-specific optimization.

**Table 2.** Different histidine kinase inhibitors, functions, target sites and limitations.

Histidine kinase inhibitor	Mode of action	Target region	Limitation	References
Luteolin	Inhibition of autophosphorylation	Catalytic ATP binding domain	Poor selectivity for histidine kinase	[67,68]
Thiazolidione	Inhibition of phosphorylation and biofilm formation	Catalytic ATP binding domain	Haemolysis induction in human erythrocytes	[69]
Benzothiazole	Inhibition of histidine kinase ADP pocket	Catalytic ATP binding domain	Cytotoxicity on eukaryotic cells	[67]
Diaryl pyrazole	Competitive inhibition of ATP binding	ATPase domain		[70]
Thiophene	Inhibition of autophosphorylation	ATP binding domain	Poor antimicrobial activity	[71]
Velikova-13	Inhibition of autophosphorylation	ATP binding domain	Poor antimicrobial effect with very low MIC	[72]
Traditional chinese medicine monomers	Inhibition of autophosphorylation	Catalytic ATP binding domain		[73]
Waldiomycin	Inhibit the autophosphorylation activities	Histidine phosphorylation domain	Moderate antibacterial activity	[74]
Maprotiline	Inhibit the binding of biofilm	Histidine kinase sensor domain	Exact molecular mechanism is unknown	[75]
Signermycin b	Inhibit the dimerization of histidine kinase	Histidine Phosphorylation Domain		[76]
LED209	Inhibited the binding of the signalling molecules	Histidine kinase sensor domain	Cytotoxicity to mammalian cells and poor selectivity	[77]
Walkmycin b and c	Inhibit the autophosphorylation activities	Cytoplasmic domains of histidine kinase		[78–80]
Diarylthiazole derivatives		Histidine kinase sensor domain	Specific mechanisms of diarylthiazole are yet to understand	[81]
Xanthoangelol b and the derivative pm-56	Target master virulence regulator (Surface proteins, release of proteases, haemolysis, leukocidins)	Phosphotransferase domain		[82]

In summary, while no HK-targeted therapeutics exist for *Neisseria* spp. yet, insights from other bacteria highlight clear structural opportunities and support the feasibility of developing TCS-directed strategies to address persistent colonization, virulence, and emerging antimicrobial resistance.

## 5. Conclusion

Two-component systems (TCSs) play a central role in Neisserial pathogenesis, antimicrobial tolerance, and host adaptation, positioning them as promising targets for next-generation therapeutics. Our analysis indicates that targeting essential TCS pathways may help overcome the limitations of conventional surface antigen–based vaccines, which are often undermined by *Neisseria* spp. high frequency of antigenic variation. For example, slipped-strand mispairing in homopolymeric tracts within capsular polysaccharide biosynthesis genes and the ability to undergo capsule switching significantly reduce the durability of vaccine-induced immunity. In contrast, TCS components and conserved regions within LOS biosynthesis pathways offer more stable and predictable targets. In parallel, emerging strategies to inhibit conserved elements of the Type IV pilus, including PilA and PilB, further complement TCS-directed approaches by disrupting Neisserial adhesion and early colonization events.

A logical next step is a multipronged strategy combining high-throughput screening with structure-guided approaches to identify small molecules that selectively target conserved TCS regions. Such efforts must also account for potential off-target effects, as bacterial histidine kinases, while distinct from human kinases, could still generate toxicity if inhibitors are insufficiently selective. The propensity for point mutations within histidine kinases also raises the possibility of rapid resistance development, underscoring the need for combination strategies or multi-target inhibitors. Furthermore, *N. meningitidis* and *N. gonorrhoea* robust immune-evasion mechanisms, including frequent antigenic variation and capsule switching, continue to complicate therapeutic design and highlight the need for approaches that act on highly conserved intracellular signaling nodes rather than surface-exposed, variable structures.

Notably, successful TCS-directed inhibitor development in other pathogens, such as histidine kinase inhibition in *Mycobacterium tuberculosis* leading to impaired cell-wall biosynthesis [[83], provides a valuable roadmap for advancing similar strategies in *Neisseria*. These precedents demonstrate the feasibility of medicinal chemistry optimization, preclinical validation, and *in vivo* toxicity testing for TCS-targeting therapeutics. Together, these insights position TCS-directed therapeutics as a realistic and urgently needed path toward combating invasive meningococcal disease and the escalating global challenge of multidrug-resistant gonorrhea.

## Data Availability

Data sharing is not applicable to this article, as no datasets were generated or analyzed during the current study.
